# Modulation of Nitrosative Stress via Glutathione-Dependent Formaldehyde Dehydrogenase and *S*-Nitrosoglutathione Reductase

**DOI:** 10.3390/ijms150814166

**Published:** 2014-08-14

**Authors:** Chuian-Fu Ken, Chih-Yu Huang, Lisa Wen, Jenq-Kuen Huang, Chi-Tsai Lin

**Affiliations:** 1 Department of Bioscience and Biotechnology and Center of Excellence for the Oceans, National Taiwan Ocean University, Keelung 202, Taiwan; E-Mail: huang_sara1208@yahoo.com.tw; 2 Institute of Biotechnology, National Changhua University of Education, Changhua 500, Taiwan; E-Mail: kencf@cc.ncue.edu.tw; 3 Department of Chemistry, Western Illinois University, Macomb, IL 61455, USA; E-Mails: L-Wen@wiu.edu (L.W.); J-Huang3@wiu.edu (J.-K.H.)

**Keywords:** *Taiwanofungus camphorata* (formerly named *Antrodia camphorata*), glutathione-dependent formaldehyde dehydrogenase (GFD), *S*-nitrosoglutathione (GSNO), *S*-nitrosoglutathione reductase (GSNOR)

## Abstract

Glutathione-dependent formaldehyde dehydrogenase (GFD) from *Taiwanofungus camphorata* plays important roles in formaldehyde detoxification and antioxidation. The enzyme is bifunctional. In addition to the GFD activity, it also functions as an effective *S*-nitrosoglutathione reductase (GSNOR) against nitrosative stress. We investigated the modulation of HEK (human embryonic kidney) 293T cells under nitrosative stress by transfecting a codon optimized GFD cDNA from *Taiwanofungus camphorata* (Tc-GFD-*O*) to these cells. The parental and transfected HEK 293T cells were then subjected to *S*-nitrosoglutathione treatment to induce nitrosative stress. The results showed that in Tc-GFD-*O*-transfected 293T cells, the expression and activity of GFD increased. Additionally, these cells under the nitrosative stress induced by *S*-nitrosoglutathione showed both higher viability and less apoptosis than the parental 293T cells. This finding suggests that the Tc-GFD-*O* in HEK 293T cells may provide a protective function under nitrosative stress.

## 1. Introduction

Oxidative stress has long been linked to neurodegenerative diseases and neurodegeneration [1,2,3]. An abnormal activation of microglia, such as caused by oxidative stresses, may become dangerous by increasing the inflammatory burden [4]. Studies using multiple antioxidants have demonstrated that reducing oxidative damage may result in a delay of neurodegenerative processes [5,6]. It is therefore important to reduce or prevent oxidative injury to the brain. One of the antioxidative mechanisms in the cell is mediated by glutathione-dependent formaldehyde dehydrogenase (GFD). GFD, an alcohol dehydrogenase 3 (ADH 3), is a bifunctional enzyme with both GFD and *S*-nitrosoglutathione reductase (GSNOR) activities. The GFD activity detoxifies formaldehyde, a highly toxic compound [7] produced in mammals by oxidative demethylation reactions [8]. Formaldehyde detoxification involves the conversion of formaldehyde to formate, which consists of three reactions [9]. The first reaction is a spontaneous condensation reaction between formaldehyde and glutathione producing *S*-hydroxymethylglutathione (HMGSH) [10]. The second reaction, catalyzed by GFD, is the oxidation of HMGSH to form *S*-formylglutathione [11]. In the third reaction, catalyzed by *S*-formylglutathione hydrolase (FGH), formate is formed and glutathione regenerated [12].

A connection between ADH3 and nitric oxide (NO) metabolism was established by Liu *et al.* (2001) [13] and Liu *et al.* (2004) [14] through studies of yeast and mouse *ADH3* knockouts. These knockout organisms are devoid of GSNOR activity, which catalyzes the NADH-dependent reduction of GSNO. The knockout organisms exhibited increased intracellular levels of GSNO and *S*-nitrosylated proteins, which, in turn, alter the activities of enzymes or structural proteins [15,16], causing alterations in tissue function. GSNO is a source of bioavailable NO, as NO is released through GSNO decomposition [17]. Nitric oxide and NO-related molecules, such as *S*-nitrosothiols (*S*-NOs), play important roles in the regulation of normal plant physiology and host defense. The ADH3 enzyme thus participates not only in the cellular homeostasis of *S*-NOs, but also in the metabolism of reactive nitrogen species and protects cells against nitrosative stress [16].

*Taiwanofungus camphorata* (*T.* *camphorata*) is a medicinal mushroom found only in the forests of Taiwan, which has traditionally been used for treating antioxidative [18] and anti-inflammatory [19] responses, among others. Recently, we established EST (expressed sequence tag) from fruiting bodies of *T.* *camphorata* in order to search for active components for possible health food applications. We have cloned and characterized several antioxidant enzymes, including a cambialistic-superoxide dismutase [20], a catalase [21], a GFD [22], a 2-Cys Prx isozyme [23] and a phospholipid hydroperoxide glutathione peroxidase [24] based on the established EST from *T.* *camphorata*.

The Tc-GFD (formerly named Ac-GFD) first reported in [22] possessed both GFD and GSNOR activities, which are believed to be responsible for formaldehyde detoxification and for fighting against nitrosative stress. In the present study, we evaluated the role of GFD in HEK 293T cells (a highly transfectable derivative of human embryonic kidney cells, which contains SV40 T-antigen) by over-expressing the Tc-GFD in these cells. Higher cell viability and less apoptosis was observed in Tc-GFD-*O*-transfected cells than the parental 293T cells. Here, we present for the first time that Tc-GFD-*O*-transfected 293T cells were protected against nitrosative stress induced by *S*-nitrosoglutathione.

## 2. Results and Discussion

### 2.1. Subcloning and Construction of Codon Optimized Tc-Glutathione-Dependent Formaldehyde Dehydrogenase (Tc-GFD-O)

**Figure 1:**
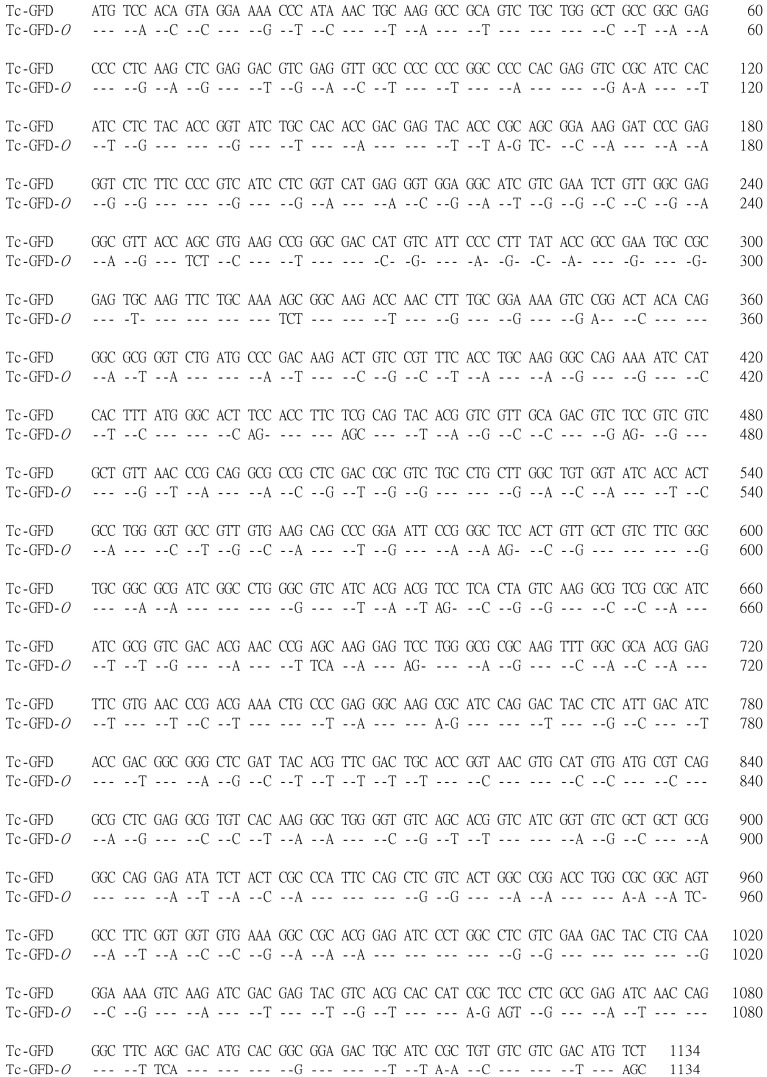
Nucleotide sequences of Tc-glutathione-dependent formaldehyde dehydrogenase(Tc-GFD) cDNA and its codon usage optimization. The codon optimized sequence of Tc-GFD shown below is designated Tc-GFD-*O*.

The codon optimization of Tc-GFD designated Tc-GFD-*O* was based on the human codon usage table and the entire sequence custom synthesized (Figure 1). The wild-type Tc-GFD or Tc-GFD-*O* was subcloned into pcDNA3 (named pcGFD or pcGFD-*O*) or pcEGFP (designated as pcGFDG or pcGFD-*O*G). The amino acid substitution mutant C47S at the active site was also constructed (designated pcC47S-*O* or pcC47S-*O*G) as described in the Materials and Methods Section.

### 2.2. Improved Viability of 293T Cells Transfected with Codon Optimized Tc-GFD-OG

**Figure 2:**
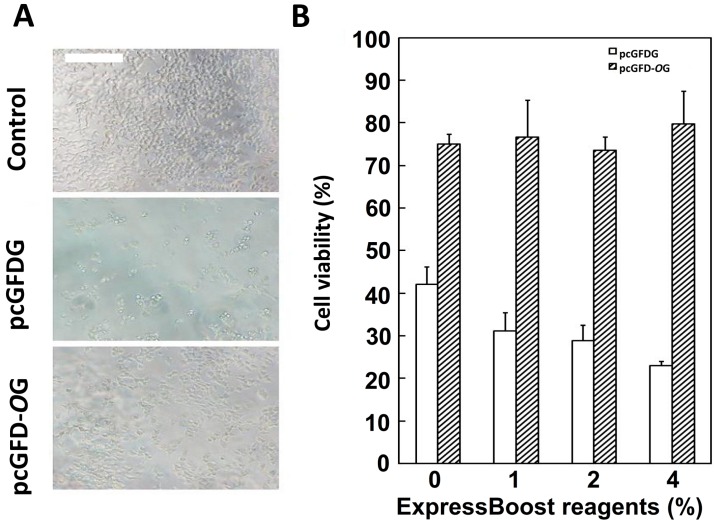
Transfection of codon optimized Tc-GFD into 293T cells improved cell viability.(**A**) 293T cells transformed to 96-well plates (1 × 10^4^ cells/well) were incubated for 48 h then transfected with pcGFDG or pcGFD-*O*G; incubation was continued for 24 h. Scale bar = 200 µm; (**B**) Different concentrations of ExpressBoost reagents were added in selected wells. After 24 h of incubation, MTT (3-(4,5-dimethylthiazol-2-yl)-2,5-diphenyltetrazolium bromide) was used to test the cell viability; the cells without plasmid were used as a control and set as 100% viability. Data are the means of three experiments.

HEK 293T cells were transfected with pcGFDG or pcGFD-*O*G. The un-transfected cells (control) had the highest cell density. Cells transfected with codon-optimized pcGFD-*O*G have higher cell density than the cells transfected with pcGFDG (Figure 2A). The transfected cells were treated with various concentrations of ExpressBoost reagents (0%–4%) to boost the protein expression of the target gene in the transfected DNA. These cells were assayed for viability/growth by MTT (3-(4,5-dimethylthiazol-2-yl)-2,5-diphenyltetrazolium bromide) assay. It is clearly shown that 293T cells transfected with pcGFD-*O*G have higher viability/growth, regardless of the amount of ExpressBoost Reagents (ATGCell Inc, Edmonton, AB, Canada) used (Figure 2B).

### 2.3. Transfection and Expression of Green Fluorescent Protein (GFP)-Fused Tc-GFD Protein and C47S Mutant in 293T

**Figure 3:**
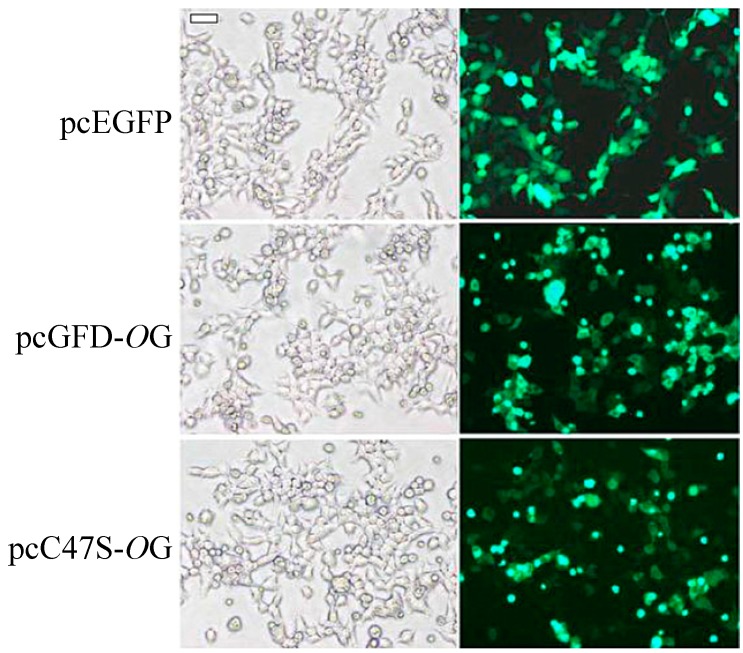
Transfection and expression of green fluorescent protein (GFP)–GFD fusion protein and its C47S mutant in 293T cells. 293T cells (6-well plate, 2.5 × 10^5^ cells/well) transfected with pcEGFP, pcGFD-*O*G and pcC47S-*O*G were incubated for 24 h. Expression of GFP-fusion protein was observed by fluorescence microscopy. Scale bar = 50 µm.

HEK 293T cells transfected with pcEGFP, pcGFD-*O*G and pcC47S-*O*G were incubated for 24 h. Expression of the green fluorescent protein (GFP)-fusion protein was observed by fluorescence microscopy. More than 50% of the cells transfected with pcEGFP (control) and pcGFD-*O*G expressed GFP and GFP–GFD fusion proteins, respectively. Cells transfected with pcC47S-*O*G have a slightly lower level of expression (Figure 3).

### 2.4. Expression of the Recombinant Tc-GFD-O in 293T Cells

**Figure 4:**
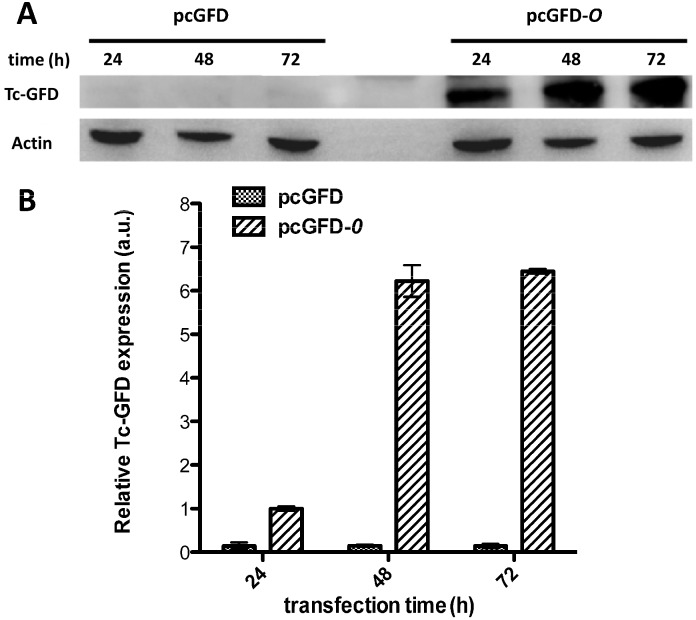
The Tc-GFD expression in 293T cells is improved by codon optimization. (**A**) At the indicated time points, cellular lysates (10 µg) of pcGFD- and pcGFD-*O-*transfected cells were analyzed by immunoblotting using anti-Tc-GFD and anti-actin antibodies; (**B**) Densitometric semi-quantification of Tc-GFD protein levels normalized to actin.

The coding region of wild-type Tc-GFD or Tc-GFD*-O* was subcloned into an expression vector, pcDNA3, as described in the Materials and Methods. Positive clones were verified by DNA sequence analysis. The amount of recombinant proteins expressed in cells transfected with pcGFD or pcGFD-*O* were compared. At the indicated time points, cellular lysates (10 µg) of pcGFD and pcGFD-*O*-transfected cells were analyzed by SDS-PAGE followed by immunoblotting using anti-Tc-GFD and anti-actin antibodies. As shown in Figure 4, pcGFD-*O*-transfected cells expressed notably high levels of Tc-GFD proteins after being transfected for 48 or 72 h, whereas pcGFD transfected cells did not expressed Tc-GFD proteins (Figure 4). The results clearly show that Tc-GFD expression in 293T cells improved greatly by codon optimization.

### 2.5. Effect of Tc-GFD Protein on S-Nitrosoglutathione (GSNO)-Induced 293T Cell Viability

**Figure 5:**
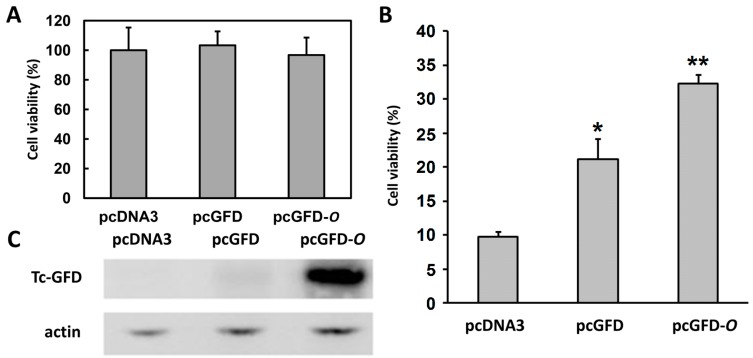
The effect of GFD and codon optimization of GFD on GSNO-induced 293T cell viability and target gene expression. (**A**) Cells were transfected with pcDNA3 (control), pcGFD or pcGFD-*O* and incubated for 40 h; (**B**) Incubation was continued in the presence of 4 mM GSNO for 8 h. Survival was assayed by the reduction of MTT. The % cell viability has been compared to the GSNO-untreated cells for each transfection condition. Data are the means of three experiments. The asterisk indicates significant difference (* *p* < 0.05), and double asterisks indicate significant difference (** *p* < 0.005) compared to the control. The data were analyzed by a paired *t*-test; (**C**) The aliquots of cell lysate (10 µg) at 40 h of transfection were analyzed by immunoblotting using anti-Tc-GFD and anti-actin antibodies.

HEK 293T cells transfected with pcDNA3 (control), pcGFD or pcGFD-*O* were incubated for 40 h. The viability of 293T cells was almost equal among the three samples at this point, as shown in Figure 5A. Incubation was continued in the presence of 4 mM GSNO for 8 h to induce oxidative/nitrosative stress. The cell viability of each transfected sample was compared to the corresponding cells before GSNO-treatment. As shown in Figure 5B, the viability of 293T cells transfected with pcGFD-*O* was higher than 293T cells transfected with pcGFD, which was higher than pcDNA3 (control). The target gene GFD expression levels were measured at 40 h transfection. Our results showed that 293T cells transfected with pcGFD-*O* expressed very high levels of Tc-GFD protein (Figure 5C), while trace amount expression was observed in pcGFD-transfected cells. Our results suggest that Tc-GFD protein improves GSNO-induced 293T cell viability/growth.

### 2.6. Effect of Mutation at C47S of GFD-O on GSNO-Induced 293T Cell Viability

**Figure 6:**
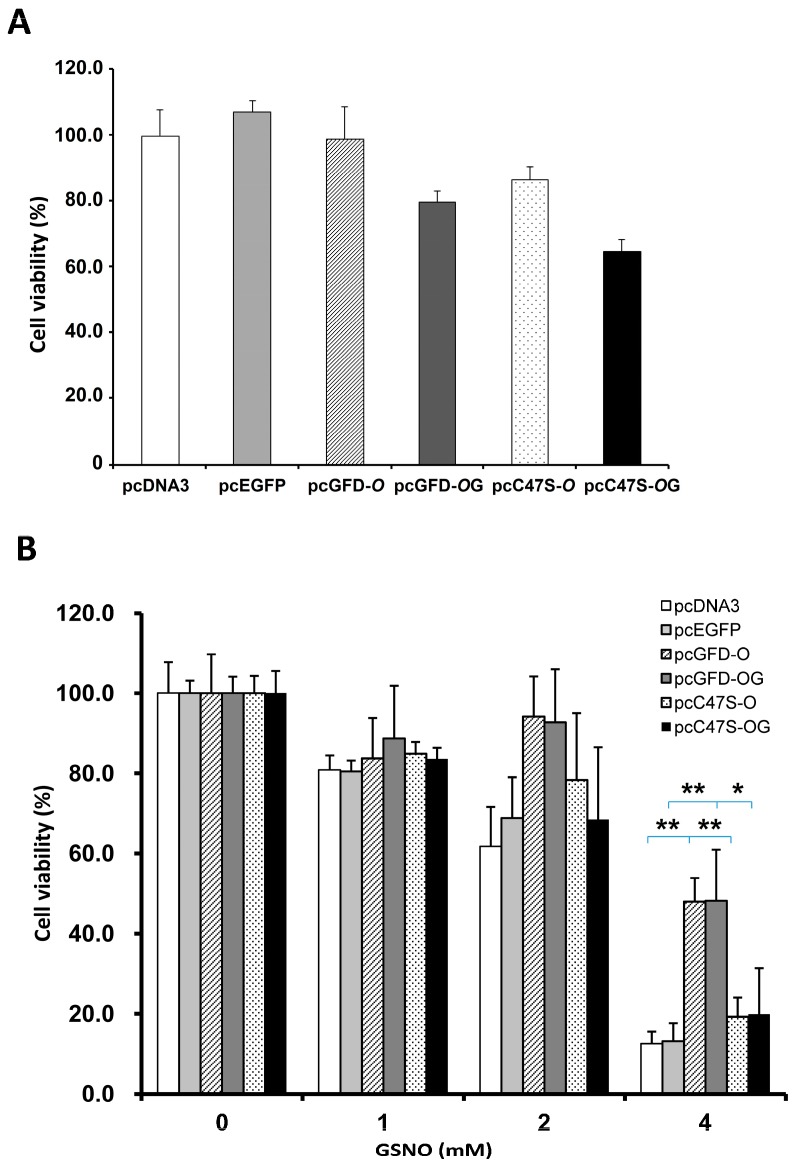
The effect of GFD on GSNO-induced 293T cell viability. 293T cells (96 wells, 1 × 10^4^ cells/well) transfected with pcDNA3, pcEGFP, pcGFD-*O*, pcGFD-*O*G, pcC47S-*O* or pcC47S-*O*G were incubated for 64 h (**A**); further incubated with 0, 1, 2, 4 mM GSNO for 8 h (**B**). Survival was assayed by the reduction of MTT. The asterisk indicates significant difference (* *p* < 0.05), and double asterisks indicate significant difference (** *p* < 0.005). The data were analyzed by a paired *t*-test.

HEK 293T cells transfected with pcDNA3, pcEGFP, pcGFD-*O*, pcGFD-*O*G, pcC47S-*O* or pcC47S-*O*G were incubated for 64 h. The relative viability of cells transfected with these plasmids is shown in Figure 6A. The transfected cells were further incubated with 0, 1, 2, 4 mM GSNO for 8 h. As shown in Figure 6B, the cell viability of 293T cells transfected with control plasmids pcDNA3 or pcEGFP decreases with increasing GSNO concentration; less than 20% of viable cells were observed at 4 mM GSNO. The viability of cells transfected with pcGFD-*O* or pcGFD-*O*G was much higher than the cells transfected with mutants of pcC47S-*O* or pcC47S-*O*G. This indicates that Cys47 is crucial to GFD activity in improving cell viability in GSNO-treated cells.

### 2.7. Effect of Tc-GFD Protein on GSNO Consumption in Transfected 293T Cells

**Figure 7:**
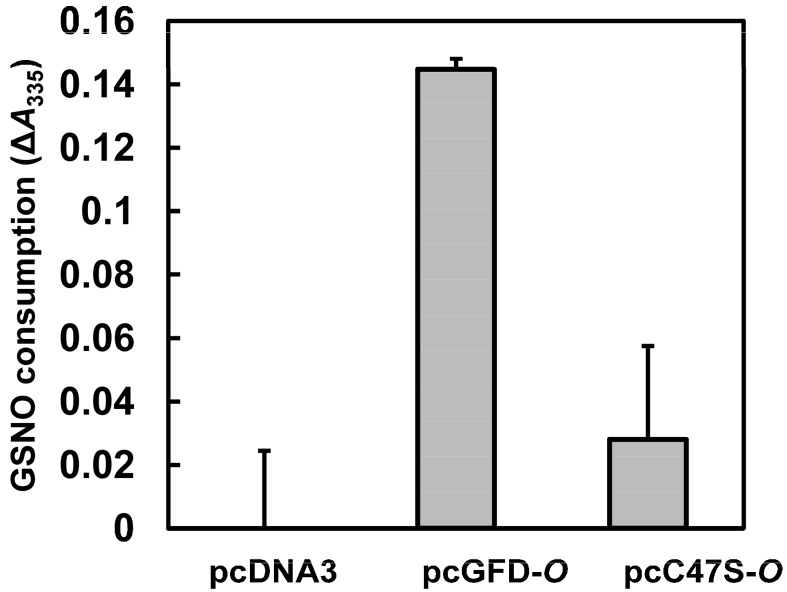
The effect of GFD on GSNO reduction. 293T cells (24 wells, 5 × 10^4^ cells/well) transfected with pcDNA3, pcGFD-*O* or pcC47S-*O* were incubated for 40 h, further incubated with 2 mM GSNO for 8 h. GSNO reduction was calculated by absorbance decrement at 335 nm (total GSNO minus the remaining GSNO amount in culture medium).

The role of Tc-GFD protein in GSNO metabolism was evaluated by measuring GSNO consumption/reduction. HEK 293T cells were transfected with pcDNA3, pcGFD-*O* or pcC47S-*O* and incubated for 40 h. The cells were treated further with 2 mM GSNO for 8 h. GSNO consumption/reduction was then measured by absorbance decrement at 335 nm. As shown in Figure 7, pcGFD-*O*-transfected cells consumed the most GSNO. The results clearly show that GSNO consumption was enhanced in the presence of Tc-GFD protein, and Cys at position 47 is crucial for the GFD activity.

### 2.8. Effect of GFD on GSNO-Induced Apoptosis in 293T Cells

**Figure 8:**
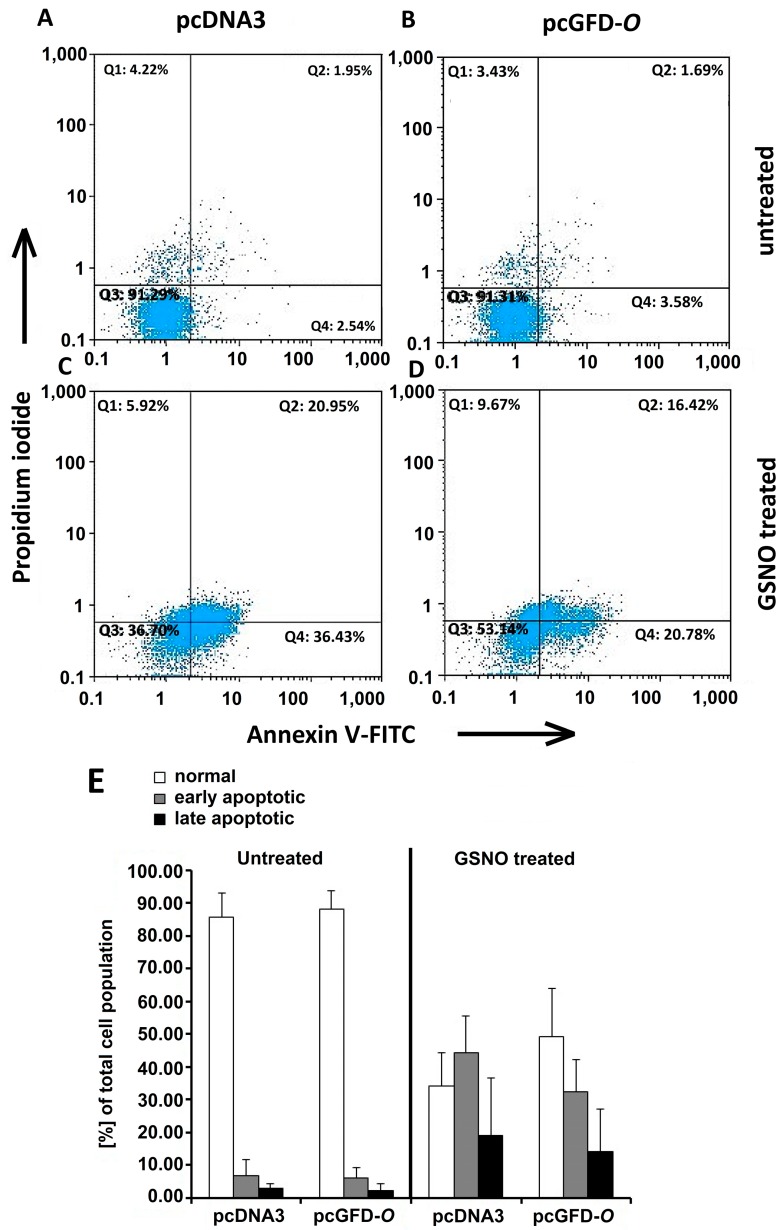
The effect of GFD on GSNO-induced embryonic kidney cell apoptosis. Embryonic kidney cells transfected with pcDNA3 and pcGFD-*O* were incubated for 40 h, further incubated with the absence (**A**,**B**) or presence (**C**,**D**) of 2 mM GSNO for 12 h; using Annexin V-FITC and PI staining and flow cytometry analysis. The lower left quadrants of each panel show the viable cells, which exclude PI and are negative for Annexin V-FITC binding. The upper right quadrants contain the non-viable, necrotic cells, positive for Annexin V-FITC binding and for PI uptake. The lower right quadrants represent the apoptotic cells, Annexin V-FITC binding positive and PI negative, demonstrating cytoplasmic membrane integrity. One representative experiment out of three is shown; (**E**) A graph comparison of cell population for each condition with standard deviations.

HEK 293T cells transfected with pcDNA3, pcGFD-*O* were incubated for 40 h. The cells were further treated in the absence or presence of 2 mM GSNO for 12 h. Apoptosis was detected using Annexin V-FITC and PI staining and flow cytometry analysis, as described in the Materials and Methods. The lower left quadrants of each panel show the viable cells, which exclude PI, and are negative for Annexin V-FITC binding. The upper right quadrants contain the non-viable, necrotic cells, positive for Annexin V-FITC binding and for PI uptake. The lower right quadrants represent the apoptotic cells, Annexin V-FITC binding positive and PI negative, demonstrating cytoplasmic membrane integrity. In our results, as shown in Figure 8, the FITC^+^/PI^−^ apoptotic cell population (early apoptotic) decreased gradually from 44.33% ± 11.05% in Panel C (pcDNA3-transfected cells and GSNO treated) to 32.30% ± 9.98% in Panel D (pcGFD-*O*-transfected cells and GSNO treated). The FITC^+^/PI^+^ necrotic cell population (late apoptotic) decreased from 18.89% ± 17.62% in Panel C (pcDNA3-transfected cells and GSNO treated) to 14.19% ± 12.93% in Panel D (pcGFD-*O*-transfected cells and GSNO treated). The results suggested that the Tc-GFD protein expressed in pcGFD-*O* transfected 293T cells had provided protection to these cells against apoptosis under GSNO induced oxidative stress.

## 3. Materials and Methods

### 3.1. Subcloning of Wild-Type Tc-GFD and Codon-Optimized GFD cDNA (Tc-GFD-O) into Mammalian Expression Vectors

A full-length GFD cDNA has been cloned from *Taiwanofungus camphorata* previously [22]. This Tc-GFD cDNA (1373 bp, EMBL Accession No. DQ396859) encodes a protein of 378 amino acid residues with a calculated molecular mass of 40,315 Da. This wild-type Tc-GFD cDNA was unable to be expressed in 293T cells. In order to improve the expression level, the GFD cDNA codons were optimized based on the human codon usage table and the entire DNA sequence synthesized (purchased from Genomics BioScience and Technology, Taipei, Taiwan). The wild-type Tc-GFD cDNA sequence and the codon-optimized sequence (Tc-GFD*-O*) are shown in Figure 1. The coding regions of the wild-type and Tc-GFD-*O* were each subcloned into pcDNA3 (mammalian expression vector with the CMV promoter, Invitrogen, Life Technologies CO., Ltd., Taipei, Taiwan), designated as pcGFD and pcGFD-*O.* The Tc-GFD-*O* coding sequence was also subcloned into pcEGFP (the vector containing green fluorescent protein) and designated as pcGFD-*O*G.

### 3.2. Site-Directed Mutagenesis of Cys 47 to Ser in Tc-GFD-O Subclones

The role of the Cys47 located in the active site of the Tc-GFD was examined by substituting the Cys with Ser in both pcGFD-*O* and pcGFD-*O*G subclones by using the Quik-Change Site-Directed Mutagenesis Kit (Stratagene, La Jolla, CA, USA). The amino acid substitution mutants are designated as pcC47S-*O* or pcC47S-*O*G. Large-scale preparation of those recombinant DNAs was performed for use in the following transfection experiments.

### 3.3. Cell Culture and Transfection of 293T Cells with Tc-GFD-O

The HEK 293T cells purchased from American Type Culture Collection are an embryonic kidney cell line immortalized by infecting the primary embryonic kidney cell cultures with a *v-raf*/*v-myc* oncogene-carrying retrovirus. 293T cells were cultured in DMEM/F-12 medium containing 10% heat-inactivated fetal bovine serum (FBS, Gibco, Life Technologies Co., Ltd., Taipei, Taiwan) at 37 °C in a humidified chamber with 5% CO_2_.

Prior to transfection, the 293T cells in 60-mm dish at about 60%–70% confluence were washed twice with serum-free medium (without antibiotic). The washed cells were then transfected with the pcDNA3, pcGFD, pcGFD-*O* or pcEGFP, pcGFD-*O*G or their mutants, pcC47S-*O* or pcC47S-*O*G, complexed with Lipofectamine (Gibco), respectively. The transfection reaction was carried out at 37 °C for 3 h. Transfected cells were grown in fresh DMEM/F-12 medium containing 10% FBS at 37 °C for at least 24 h before further experimentation. Cells transfected with pcDNA3 or pcEGFP DNA were used as a control.

### 3.4. Cell Viability Assay

Cell viability was determined by the MTT assay [25]. The assay is based on the cleavage of the yellow MTT to purple formazan crystals by metabolic viable cells. After removal of the conditioned media from the plates, the cells were incubated with 0.5 mg/mL MTT (100 µL/well) for 4 h. Next, the supernatants were aspirated, and the insoluble formazan product was dissolved in dimethyl sulfoxide (DMSO) (100 µL/well) for 1 h. The extent of formazan production reflecting cell viability was quantified by measuring the absorbance at 595 nm.

### 3.5. Expression of Tc-GFD Protein in 293T Cells

HEK 293T cells (4 × 10^5^ cells in a 10-mL/10-cm dish) were transfected with 4 µg of each plasmid (pcDNA3, pcGFD, pcGFD-*O*) and cultured for 24, 48, or 72 h. At the indicated time points, cellular lysates (10 µg) of pcGFD and pcGFD-*O*-transfected cells were analyzed by SDS-PAGE followed by immunoblotting (One-Step™ Western Kit, GenScript, Piscataway, NJ, USA) using anti-Tc-GFD (a custom antibody produced by Yao-Hong Biotechnology, Taipei, Taiwan) and anti-actin antibody (purchased from Yao-Hong Biotechnology, Taiwan). The Tc-GFD protein expressed in each transfected cell was quantified by a densitometer (Molecular Dynamics, Modesto, CA, USA) and normalized to the level of actin, a housekeeping protein, whose levels should be fairly constant.

### 3.6. Induction of Oxidative/Nitrosative Stress in Parental and Transfected 293T Cells by S-Nitrosoglutathione Treatment

*S*-Nitrosoglutathione (GSNO) has been shown to induce oxidative/nitrosative stress in *Drosophila melanogaster* [26]. Thus, GSNO was used to induce oxidative/nitrosative stress in 293 cells in this study. HEK 293T cells transfected with pcDNA3 (control), pcGFD or pcGFD-*O* were incubated for 40 h. Incubation was continued in the presence of 4 mM GSNO for 8 h. Survival/growth of the cells was evaluated by the MTT assay. To quantify the expression levels of Tc-GFD proteins after 40 h of transfection, aliquots of each transfected cell lysates (10 µg) were analyzed by SDS-PAGE followed by immunoblotting using anti-Tc-GFD and anti-actin antibodies.

### 3.7. Transfection Efficiency of GFD in 293T Cells

HEK 293T cells (6-well plate, 2.5 × 10^5^ cells/well) transfected with pcEGFP, pcGFD-*O*G and pcC47S-*O*G were incubated for 24 h. Expression of GFP-fusion proteins in the transfected 293T cells was observed by fluorescence microscopy.

### 3.8. Effect of Tc-GFD Protein on GSNO-Induced 293T Cell Viability

HEK 293T cells (96-well plate, 1 × 10^4^ cells/well) transfected with pcDNA3, pcEGFP, pcGFD-*O*, pcGFD-*O*G, pcC47S-*O* or pcC47S-*O*G were incubated for 64 h. Incubation was continued with 0, 1, 2, 4 mM GSNO for 8 h. Survival/growth was evaluated by the MTT assay.

### 3.9. Effect of pcGFD-O or pcC47S-O on GSNO Consumption

HEK 293T cells (24 well plate, 5 × 10^4^ cells/well) transfected with pcDNA3, pcGFD-*O* or pcC47S-*O* were incubated for 40 h. Incubation was continued with 2 mM GSNO for 8 h. The concentration of GSNO in these cells was determined by its absorbance at 335 nm and calculated using the extinction coefficient of 767 M^−1^·cm^−1^ [27].

### 3.10. Effect of GFD on GSNO-Induced 293T Cell Apoptosis

HEK 293T cells transfected with pcDNA3, pcGFD-*O* were incubated for 40 h. Incubation was continued in the absence or presence of 2 mM GSNO for 12 h. Apoptosis induced by GSNO was detected by flow cytometry using a combination of cytofluorometric stains: Annexin V-FITC and propidium iodide (PI) (Annexin V-FITC Apoptosis Detection Kit, Strong Biotech, Taipei, Taiwan). Annexin V conjugated with fluorescein isothiocyanate (FITC) was used to label phosphatidylserine on the membrane surface of apoptotic cells [28]. The PI stain was to differentiate apoptotic cells from viable and necrotic cells. It is impermeant to live cells, but can enter dead cells easily, where it binds to nucleic acids and become fluorescent. The combination of both dyes allows the differentiation among viable cells (Annexin V negative, PI negative), early apoptotic cells (Annexin V positive, PI negative), and necrotic cells (Annexin V positive, PI positive) [28].

## 4. Conclusions

The GFD recombinant enzyme from *Taiwanofungus camphorata* has been demonstrated to be responsible for formaldehyde detoxification and fighting against nitrosative stress [22]. These characteristics make it a good candidate for transfection purpose. In this study, we have demonstrated that when the codons optimized sequence (Tc-GFD*-O*) were transfected to 293T cells, GFD recombinant enzyme was highly expressed. The expressed Tc-GFD protein has been shown to improve the viability of the transfected 293T cells. Additionally, the protein showed improved GSNO-induced 293T cell viability/growth and protected the cells against apoptosis under oxidative stress (GSNO treated). The mechanism by which Tc-GFD exhibits a protective function against oxidative stress may be due to its ability to enhance GSNO consumption (Figure 7). Our results may provide an alternative approach to the development of a protection strategy against nitrosative stress. In addition, this study may provide a rationale for the development of treatments for neurodegenerative diseases, such as prion’s, Alzheimer’s, Parkinson’s and Huntington’s diseases. One of the major neuropathological changes of these diseases is defined by the neuronal cell apoptosis [1,2,3,5,6].
